# Physical activity intervention for rural middle-aged and older Australian adults: a pilot implementation study of the *ecofit* program delivered in a real-world setting

**DOI:** 10.1186/s40814-021-00823-1

**Published:** 2021-03-24

**Authors:** Magdalena Wilczynska, Anna K. Jansson, David R. Lubans, Jordan J. Smith, Sara L. Robards, Ronald C. Plotnikoff

**Affiliations:** Priority Research Centre for Physical Activity and Nutrition, University of Newcastle, Callaghan, Newcastle, NSW 2308 Australia

**Keywords:** Smartphone application, Physical activity, Outdoor environment, Older adults

## Abstract

**Background:**

*ecofit* is an evidence-based multi-component physical activity intervention that integrates smartphone technology, the outdoor environment, and social support. In a previous efficacy trial, significant improvements were found across several clinical, fitness, and mental health outcomes among adults at risk of (or with) type 2 diabetes.

**Methods:**

The aim of the present pilot study was to evaluate a number of patient-centered and feasibility outcomes of the *ecofit* intervention in a “real-world” setting, using a scalable implementation model. *ecofit* was adapted and implemented by a rural municipal council in the Upper Hunter Shire, New South Wales, Australia, and evaluated using a single-group pre-post design. Inactive middle-aged and older adults (*N*=59) were recruited and assessed at 6 (primary time-point) and 20 weeks (follow-up).

**Results:**

Improvements were found in this predominantly overweight and obese sample for aerobic fitness, functional mobility, upper and lower body muscular fitness, systolic blood pressure, and waist circumference at 6 weeks. At 20 weeks, effects were found for aerobic fitness, functional mobility, upper and lower body muscular fitness, and systolic blood pressure. Overall, participants were satisfied with the *ecofit* program. Participants attended the 6-week primary time-point (66.1%) and follow-up at 20 weeks (41.6%).

**Conclusions:**

Our findings support the preliminary effectiveness and feasibility of the *ecofit* intervention delivered by municipal council staff following a brief training from the research team. This study provides valuable preliminary evidence to support a larger implementation trial.

## Key messages regarding feasibility

- What uncertainties existed regarding the feasibility?
  Can the ecofit program be delivered in a real world setting with minimal support from the university research team?
- What are the key feasibility findings?
  The program included retention and participant satisfaction components and was able to be delivered by non-researchers (i.e., municipal staff).
- What are the implications of the feasibility findings for the design of the main study?
  The results from this study provided valuable preliminary evidence to support a larger implementation trial. Future *ecofit* implementation studies should implement quality assurance protocols (e.g., to assess the fidelity of program delivery), gather more feasibility data (e.g., adherence and reasons for dropouts), and implement strategies to recruit more males.

## Background

Participation in regular physical activity is associated with reduced risks of cardiovascular disease; overweight/obesity; type 2 diabetes mellitus (T2DM); numerous cancers; mental, musculoskeletal, and reproductive health problems; and reduced falls risk in elderly [1–4]. In addition, higher levels of physical activity have been linked to enhanced social and psychological functioning, including reduced anxiety, depression, and stress [5]. Despite the benefits, 50% of adults (aged 18–64) and 75% of older adults (65 years and over) in Australia do not accrue enough physical activity [6].

The present study follows on from the original *ecofit* efficacy trial, which is a multi-component community-based physical activity intervention that integrates smartphone technology, the outdoor environment, and social support [7, 8]. This randomized controlled trial targeted adults at risk of, or diagnosed with, type 2 diabetes who were not meeting physical activity guidelines. At the 10-week primary time-point, the study found significant effects for aerobic fitness, physical activity, upper and lower body muscular fitness, functionality, waist circumference, and systolic blood pressure [7]. Most of these effects were sustained at the 20-week follow-up (i.e., aerobic fitness, upper and lower body muscular fitness, functional mobility, systolic blood pressure, waist circumference, and depression symptoms) [7].

Following the success of the efficacy trial, the aim of the current study was to conduct a pilot evaluation of the *ecofit* intervention using a scalable implementation model (i.e., municipal council delivery) among inactive middle-aged and older adults residing in an Australian rural community. As such, the aim of the present pilot study was to evaluate the preliminary effectiveness and feasibility of the *ecofit* intervention in a “real-world” setting, using a scalable implementation model.

The main objectives for this trial were as follows:

1. To assess the preliminary effectiveness of the *ecofit* intervention on a number of patient-centered outcomes (i.e., aerobic fitness, functional mobility, upper and lower body muscular fitness, systolic and diastolic blood pressure, and waist circumference)
2. To assess retention of participants at 6 weeks and at 20 weeks
3. To evaluate the different components of the *ecofit* program (i.e., cognitive mentoring sessions, outdoor personal training sessions, outdoor fixed fitness equipment, and the smartphone app)

## Methods

### Study design

This pilot implementation study involved a pre-post experimental research design. The 20-week study took place in August 2017 and was based on the *ecofit* efficacy trial, protocol published elsewhere [8]. The *ecofit* program was adapted and implemented by health officers employed by the Upper Hunter Shire Council, New South Wales (NSW), Australia. The Upper Hunter Shire Council is a local government area which consists of four rural communities with an estimated population of 10,507 of which, 4698 (44.7%) are 45 years of age or older [9]. Assessments were conducted at baseline, 6 weeks and 20 weeks post-baseline.

### Participants

Participants were recruited by the Upper Hunter Shire Council using a variety of strategies (e.g., local radio stations, flyers, newspaper advertisements and local seniors’ clubs). Inclusion criteria included (i) ≥45 years of age and (ii) not meeting current physical activity guidelines. Participants were excluded if they had a medical condition that might preclude participation in physical activity. All participants provided written informed consent prior to enrollment.

### Intervention components

The face-to-face sessions were adopted from the *ecofit* efficacy trial [8] and were composed of two parts; cognitive mentoring (30 min) followed by an outdoor exercise session (60 min). The cognitive mentoring sessions were developed to provide participants with skills and strategies to overcome barriers, increase motivation, and set goals. The supervised outdoor training sessions were developed to provide participants with the confidence, skills, and knowledge to perform aerobic and resistance activities using the outdoor built environment (e.g., stairs, railings, benches). Sessions were composed of approximately 50% aerobic and 50% resistance training with a moderate-to-vigorous intensity equal to (or greater) than three metabolic equivalents. The aerobic workout included approximately 3 km of moderate-to-vigorous intensity aerobic activity (i.e., walking or jogging) and the resistance workout included six exercises (i.e., abdominal strengthening, external rotations, knee lifts, pulls-ups, push-ups, and squats). Participants were also provided with the *ecofit* smartphone app for the duration of the study. The app had been adapted from the *ecofit* efficacy trial [8] and included tailored workouts designed specifically for four locations in the Upper Hunter Shire, a rural area of NSW.

### Intervention overview

The intervention consisted of two phases; phase 1 (1–6 weeks) and phase 2 (7–20 weeks). During phase 1, participants attended the face-to-face session once per week***.*** Phase 2 consisted of three parts. In the first part (weeks 7–10), participants received no face-to-face sessions but were encouraged to meet with other participants to continue their workouts. Participants were then provided with weekly face-to-face sessions for a further 4 weeks (weeks 11–14); this was followed by 6 weeks of no sessions (weeks 15–20). For the duration of the project, participants had access to the purpose-built *ecofit* smartphone application.

A training day was held at the University of Newcastle for the two Council representatives who would conduct the assessments and deliver the intervention. One of the Council representatives was a qualified nurse and personal trainer and was the main person who implemented the program. The other Council representative was a Council worker who had a personal interest in physical activity promotion and assisted with the delivery of the program. A “training the trainer” approach was utilized and included an 8-hour workshop where the Council representatives were trained by the original *ecofit* research team in how to deliver the program. The content of the program was adopted from the original study [7, 8]. This training included instructions and guidance on how to conduct the face-to-face components (i.e., the outdoor exercise and cognitive mentoring sessions) and protocols for data collection and participant outcomes. Some of the materials (i.e., face-to-face session and in the app) were adopted to reflect this older population. For example, a greater focus was provided on physical activity barriers such as joint pain, balance issues, and lack of company as opposed to a lack of time or work fatigue. The Council representatives were provided with a training manual, which included written material and slides for the face-to-face session, and a standardized assessment manual for data collection. After the training session, the Council representatives independently implemented the *ecofit* program across the four locations, i.e., created recruitment material, commenced recruitment, undertook assessments, and ran and organized the training and cognitive mentoring sessions with no involvement from the research team.

### Outcomes

Assessments were conducted at baseline, 6 weeks (primary time-point) and at 20 weeks (follow-up). Baseline assessments were conducted prior to commencing in the *ecofit* implementation program. Participants were measured using most (but not all) measures from the *ecofit* efficacy trial [8]. In addition, the present study used the 6-Minute Walk Test [10] instead of the Single Stage Treadmill Walking test to measure aerobic fitness [11]. All assessments were administered by the trained Upper Hunter Shire Council representatives. Upon completion of assessments, data log sheets were returned to the researchers at the University of Newcastle for analysis.

#### Patient-centered outcomes

##### Aerobic fitness

Aerobic fitness was measured using the 6-Minute Walk Test [12]. The 6-Minute Walk Test has been extensively used in both clinical and research contexts to measure cardiorespiratory fitness [10]. In preparations for the test, a 30-metre straight track was set up by the assessors. During the test, participants were instructed to complete as many laps (one lap constituted walking back and forth along the track) as possible for 6 minutes.

##### Functional mobility

Functional mobility was assessed using the Timed Up and Go Test [13]. The Timed Up and Go Test assess an individual’s mobility and static and dynamic balance. During the test, participants starts from a seated position, and on command stands up from the chair and walks to a point 3 metre from the chair. Once the participant has reached the 3 metre mark, they turn around and walk back to the chair and sits down. Timing starts when participants stand up from the chair and ends when they return to the chair.

##### Upper body muscular fitness

Upper body muscular fitness was assessed with the Arm Curl Test [14]. During the Arm Curl Test, participants were instructed to while seated perform as many arm curls as possible in 30 seconds.

##### Lower body muscular fitness

Lower body muscular fitness was assessed using the chair stand test [14]. The testing procedures include participants starting from a seated position on a chair and standing up and sitting down (one repetition) as many times as possible within 30 seconds. The participant’s score is the number of repetitions completed within 30 seconds [15].

##### Blood pressure

Systolic and diastolic blood pressure was measured using a standard digital automatic blood pressure monitor.

##### Waist circumference

Waist circumference was measured between the top of the iliac crest and the lowest floating rib using a non-extensile steal tape. Measures were recorded to the nearest 0.1 centimeter.

#### Feasibility outcomes

##### Retention

Retention rates were calculated by the number of participants completing the assessments at each assessment time-point (6 weeks and 20 weeks) compared to baseline.

##### Program satisfaction

A process evaluation survey was provided to all participants who attended their final follow-up assessment. The survey was divided up in two parts (i.e., part 1 and part 2). Part 1 included close-ended questions regarding the cognitive mentoring sessions, outdoor personal training sessions, outdoor fixed fitness equipment, and the smartphone app. Response options for all close-ended questions ranged from 1 (strongly disagree) to 5 (strongly agree), or 1 (poor) to 5 (excellent). In part 2 of the survey, participants were asked to comment (i.e., likes and dislikes) on each of the abovementioned components.

### Sample size

The Upper Hunter Shire Council was solely in charge of recruitment based on their contextual and pragmatic factors; therefore, no formal sample size calculation was performed. Effect sizes are however reported to gain an understanding of the magnitude of the intervention effects

### Statistical methods

Statistical analysis of the study outcomes was conducted using IBM SPSS Statistics, Version 22.0. Results are reported as means (*M*) and standard deviation (SD). Effect sizes were calculated for all health-related outcomes at 6 weeks and 20 weeks using Cohen’s *d*. Two paired-sample *t*-tests were conducted to compare health-related outcomes between baseline and 6 weeks, and between baseline and 20 weeks. Corresponding 95% confidence intervals were calculated for mean paired differences (paired sample *t*-tests). Independent sample *t*-tests and descriptive statistics were also performed to compare available participant characteristics (i.e., age, sex, and BMI) between participants attending assessments at 6 weeks and 20 weeks. Descriptive statistics were used to summarize participants’ demographics. Due to the small sample size, the results in this study should be interpreted with caution.

## Results

### Study sample

The demographic characteristics of the sample consisted of the following; *M* (age)= 62.3 years (*SD* = 11.58; *min = 50, max = 82*), 95% females, *M* (body mass index (BMI)) = 30.68 (*SD* = 6.3) with 47% and 38% classified as obese and overweight respectively [16].

### Patient-centered outcomes

The results from the 6-week primary time-point are presented in Table 1. After 6 weeks, statistically significant (*p* <.05) improvements were observed for aerobic fitness (*M* = 3.59; 95% CI 2.44, 4.74, *d* = 1.01), functional mobility (*M* = −1.74; 95% CI −2.21, −1.27, *d* = −1.23), upper body (*M* = 2.71; 95% CI 1.33, 4.08, *d* = 0.65) and lower body (*M* = 2.15; 95% CI 1.21, 3.09, *d* = .74) muscular fitness, systolic blood pressure (*M* = −7.37; 95% CI −13.62, −1.11, *d* = −.39), and waist circumference (*M* = −3.49; 95% CI −5.15, −1.83, *d* = −.68). There was no statistically significant (*p* > .05) improvement for diastolic blood pressure.

**Table 1:** Results at the 6-week primary time-point *(s)* seconds, *(l)* laps

Outcomes	Baseline M (SD)	6 weeks M (SD)	Difference between groups M (95% CI)	***n***	***t***	***p*** value	Effect size (Cohen’s ***d***)
6-Minute Walking Test (l)	14.89 (3.47)	18.48 (2.58)	3.59 (2.44, 4.74)	39	6.33	**<.001**	1.01
Timed Up and Go test (s)	7.73 (1.58)	5.98 (1.43)	−1.74 (−2.21, −1.27)	38	−7.54	**<.001**	−1.23
Arm Curl Test (reps)	18.66 (3.53)	21.37 (3.48)	2.71 (1.33, 4.08)	38	4.00	**<.001**	0.65
Chair stand test (reps)	13.41 (3.22)	15.56 (2.63)	2.15 (1.21, 3.09)	39	4.63	**<.001**	0.74
Systolic blood pressure (mm Hg)	142.47 (22.89)	135.11 (18.98)	−7.37 (−13.62, −1.11)	38	−2.38	**.022**	−0.39
Diastolic blood pressure (mm Hg)	80.26 (10.18)	79.45 (10.43)	−.81 (−2.11, 3.74)	38	−0.56	.576	−0.09
Waist circumference (cm)	99.66 (15.35)	96.17 (13.70)	−3.49 (−5.15, −1.83)	39	−4.25	**<.001**	−0.68

The results from the 20-week follow-up time-point are presented in Table 2. At 20-week follow-up, statistically significant (*p* < .05) improvements were observed for aerobic fitness (*M* = 3.88; 95% CI 2.49, 5.27, *d* = 1.31), functional mobility (*M* = −.90; 95% CI −1.34, −.46), *d* = −.87), upper body (*M* = 5.15; 95% CI 3.72, 7.03, *d* = 1.19) and lower body (*M* = 3.93; 95% CI 2.55, 5.32, *d* = 1.20) muscular fitness, and systolic blood pressure (*M* = −12.70; 95% CI −19.80, −5.60, *d* = −.76). There were no statistically significant differences (*p* > .05) for diastolic blood pressure or waist circumference.

**Table 2:** Results at the 20-week time-point

Outcomes	Baseline M (SD)	20 weeks M (SD)	Difference between groups M (95% CI)	***n***	***t***	***p v***alue	Effect size (Cohen’s ***d***)
6-Minute Walking Test (l)	14.61 (2.97)	18.5 (2.01)	3.88 (2.49, 5.27)	20	5.85	**<.001**	1.31
Timed Up and Go Test (s)	7.55 (1.61)	6.64 (1.26)	−0.90 (−1.34, −0.46)	24	−4.22	**<.001**	−0.87
Arm Curl Test (reps)	18.78 (3.42)	23.93 (3.68)	5.15 (3.27, 7.03)	23	5.68	**<.001**	1.19
Chair stand test (reps)	13.27 (4.06)	17.21 (4.18)	3.93 (2.55, 5.32)	24	5.88	**<.001**	1.20
Systolic blood pressure (mm Hg)	146.04 (24.10)	133.33 (13.48)	−12.70 (−19.80, −5.60)	24	−3.70	**<.001**	−0.76
Diastolic blood pressure (mm Hg)	83.25 (9.52)	80.29 (9.07)	−2.95 (−6.77, 0.86)	24	−1.60	.123	−0.33
Waist circumference (cm)	99.03 (15.31)	96.69 (12.25)	−2.34 (−5.43, 0.75)	24	−1.56	.132	−0.32

*(s)* seconds, *(l)* laps

### Feasibility outcomes

In total, 39 (66.1%) participants attended their 6-week assessment and 24 participants (41.3%) attended their 20-week follow-up assessments. The following was recorded for the 24 participants who completed the process measures. Nearly all of the participants (91.3%) agreed/strongly agreed they were satisfied with the overall *ecofit* program in providing useful information and skills about how to be physically active. The majority (77.3%) indicated the cognitive mentoring was good/excellent and all (100%) specified the personal training sessions were good/excellent. Most participants agreed/strongly agreed the fixed outdoor equipment was easy to use (93.8%) and of good quality (94.1%). Approximately half (52.6%) of the participants agreed/strongly agreed the app increased their knowledge of how to use the outdoor physical environment to be more active. Moreover, 41.2% agreed/strongly agreed that the app helped participants monitor their physical activity progress while 26.3% agreed/strongly agreed the app helped set goals and plan their physical activity. Of note, most participants reported to the municipal council representative that they had not been able to use the smartphone app due to poor internet connection. Adherence to the scheduled face-to-face personal training sessions was not collected by the facilitators.

### Loss to follow-up

At the 6-week time-point, there was no statistical significant difference in age (reported at baseline) between attenders (*M* = 63.5, SD = 8.3) and non-attenders (*M* = 59.8, SD = 16.6; *p* > 0.05). Likewise, there was no statistical significant difference in BMI (measured at baseline) among those who attended the 6-week time-point (*M* = 29.9, SD = 6.0) compared to those who did not (*M* = 32.6, SD = 7.1; *p* > 0.05). No males (*n* = 2) attended the 6-week time-point.

There was no statistical significant difference in age (reported at baseline) between attenders (*M* = 63.0, SD =8.0) and non-attenders (*M* =61.8, SD =13.8) at the 20-week time-point (*p* > 0.05) or for BMI (measured at baseline) between attenders (*M* = 30.2, SD = 5.3) and non-attenders (*M* = 31.4, SD = 7.2; *p* > 0.05). At the 20-week follow-up, one male participant (out of two) attended the assessment.

## Discussion

The aim of the present pilot study was to evaluate the preliminary effectiveness and feasibility of the *ecofit* intervention in a “real-world” setting, using a scalable implementation model. We found improvements in most health-related outcomes at 6 weeks and 20 weeks. Participants were overall satisfied with the *ecofit* program; however, study retention was relatively low (66.1% at 6 weeks and 41.3% at 20 weeks).

Overall, these results are in line with the efficacy trial which reported similar effects [7]. However, it should be noted that the magnitude of the effect sizes was smaller than those reported in the efficacy trial. This may be explained by the smaller sample in the present study and/or the “voltage drop” of the intervention dose in the real-life delivery setting where the fidelity of the program may have been compromised. Meeting both the aerobic and the muscle strengthening physical activity guidelines among this population has been associated with many physiological, psychological, and clinical benefits [1, 2]. Thus, the results from this study are promising given the prevalence of meeting the physical activity guidelines is very low among this population age group [6].

People were satisfied with the program; however, many participants reported not using the app due to poor Internet connection. Indeed, using web-based technology may prove problematic in rural areas due to poor internet services, and/or poor technological literacy among older adults. This is an important consideration for future studies that plan to carry out web-based interventions in rural areas. Given the low levels of app usage, our findings suggest that the face-to-face component may have been sufficient to improve outcomes among this group (and indeed may be preferable). Furthermore, only people who attended the 20-week assessment (41.3%) completed the process evaluation survey and many participants did not use the app; thus, the results may be biased and should be interpreted with caution.

At both 6 and 20 weeks post-baseline, there were no statistically significant differences in age and BMI between those that attended the assessments and those that did not (*p* > .05). This suggests that in this sample, age and BMI were not determining factors as to whether participants remained in the program or not. However, there may be other participant characteristics and/or demographical information that may have been statistically different between attenders and non-attenders which this study was unable to assess. Our sample had very few males (*n* = 2); of these, none attended the 6-week assessment whereas one male attended their 20-week assessment. As such, given that it remains unknown why participants dropped out of the study, the results should be interpreted with caution, particularly when generalizing the results to males in this subpopulation.

Study retention at 20 weeks was relatively low (41.3% at follow-up). It remains unclear why participants dropped out of the study as this information was not documented by the council representatives. In addition, we were unable to assess other feasibility components (i.e., recruitment details and program adherence) as these were also not recorded. Further, while the council representatives were provided training in data collection, we acknowledge that there was no quality assurance process in place during the data collection periods. As such, the results should be interpreted with caution. Future *ecofit* studies may need to consider effective retention strategies such as encouraging community ownership, offering incentives to participants, and/or providing personalized support (i.e., monitoring/checking-in strategies) [17]. In addition, it is recommended to provide rigorous training and ensure the protocol manuals and data collection sheets are easy to use for non-researchers. It is also suggested to implement a quality assurance protocol to ensure validity and reliability during the data collection process.

While many physical activity interventions have proven effective in controlled research settings, few studies to date have been conducted in “real world” environments [18]. Indeed, successful translation and maintenance of efficacious physical activity interventions is complex and challenging, and few successful examples appear in the published literature. For intervention strategies to shift populations to be more active, joint efforts between researchers, government agencies, and the general community are essential for the “scale-up” of efficacious interventions [18]. Thus, municipal local councils are in ideal positions to assist with physical activity promotion as one of their main objectives are to promote health and well-being among residents through the provision of facilities [19]. This study provides support for the *ecofit* project to be operationalized and effectively delivered under limited supervision from researchers, with effective results and participant program satisfaction. Further cost-effective analysis of *ecofit* would be useful, which is being conducted in a current large randomized controlled effectiveness trial [20].

The main strength of this study is the implementation of an efficacious program in a real-world context with limited involvement from the researchers. Another strength is the design of the program. The *ecofit* program only requires simple infrastructure (i.e., railings, stairs, benches) and thus can be adapted to most outdoor locations. Limitations of the study include not employing an intention-to-treat analysis, the lack of male study participants, and not assessing recruitment efficacy. The challenge in engaging males is consistent with previous community-based physical activity studies [21, 22]. Another limitation is the loss of sample at the 20-week follow-up and not noting the reason for drop-out. The high drop-out rate may be explained by a number of reasons, including not providing incentives to participants at assessment time-points, the time and/or effort burden to participate in the research evaluation component, or that participants failed to engage and/or did not experience a positive experience from participating in the *ecofit* program. This research, however, will hopefully guide researchers and practitioners in the design and implementation of practical programs which target the growing overweight and obese populations.

## Conclusions

This was the first attempt to deliver the *ecofit* intervention outside a research setting in a rural context. Results from the present study support the preliminary effectiveness and feasibility of the *ecofit* intervention and will assist in the design of a larger implementation trial.

## Data Availability

The datasets generated or analyzed during the current study are not publicly available. They are available from the corresponding author on reasonable request.
